# Draft Genome Sequence of “*Halomonas chromatireducens*” Strain AGD 8-3, a Haloalkaliphilic Chromate- and Selenite-Reducing Gammaproteobacterium

**DOI:** 10.1128/genomeA.00160-16

**Published:** 2016-03-17

**Authors:** Fedor S. Sharko, Anna A. Shapovalova, Svetlana V. Tsygankova, Anastasia V. Komova, Eugenia S. Boulygina, Anton B. Teslyuk, Pavel M. Gotovtsev, Zorigto B. Namsaraev, Tatiana V. Khijniak, Artem V. Nedoluzhko, Raif G. Vasilov

**Affiliations:** a Research Center of Biotechnology of the Russian Academy of Sciences, Moscow, Russia; b National Research Centre “Kurchatov Institute,” Moscow, Russia

## Abstract

Here, we report the complete genome sequence (3.97 Mb) of “*Halomonas chromatireducens*” AGD 8-3, a denitrifying bacterium capable of chromate and selenite reduction under extreme haloalkaline conditions. This strain was isolated from soda solonchak soils of the Kulunda steppe, Russian Federation.

## GENOME ANNOUNCEMENT

“*Halomonas chromatireducens*” strain AGD 8-3, which is capable of denitrification and chromate and selenite reduction under extreme haloalkaline conditions, was isolated from soda solonchak soils of the Kulunda steppe (Russian Federation) (1). DNA was extracted using the protocol described before by Marusina and colleagues (2). A paired-end DNA library (average insert size 325 bp) was constructed using the NEBNext DNA library prep reagent set for Illumina (New England Biolabs, USA). The DNA library was sequenced using an Illumina HiSeq 1500 platform (Illumina, USA) with 150-bp paired-end reads (458,872 Illumina paired-end reads were generated). The error correction of the Illumina reads was conducted using the latest version of the SOAPdenovo2 correction tool (3); the reads were then merged (up to 68%) using Pear software (4). SPAdes software was used for *de novo* assembly of the merged Illumina reads (5). As a result, 247 contigs were assembled (*N*_50_ = 66,254 bp). The draft genome sequence of *H. chromatireducens* AGD 8-3 was constructed using the Python script CONTIGuator (6), with the previously sequenced *Halomonas elongata* genome (NC_014532.1) (7) as a reference. The GapCloser tool from the SOAPdenovo2 assembler (3) was used to close the gaps emerging during the *de novo* genome assembly. The genome size is estimated to be 3,973,651 bp with a G+C content of 62.8%.

According to the genome sequence, *H. chromatireducens* AGD 8-3 is taxonomically closely related to the halophilic gammaproteobacterium *H. elongata* DSM 2581 and the moderately halophilic gammaproteobacterium *Chromohalobacter salexigens* DSM 3043, which were sequenced previously (7, 8). Gene prediction was performed using RAST (9) (http://rast.nmpdr.org), which produced 3,680 protein-coding sequences. Generally, the genome sequences of *H. chromatireducens* and its closest relatives have striking differences: approximately 12% nucleotide differences with the whole-genome sequence of *H. elongata* were found.

The complete genome of *H. chromatireducens* AGD 8-3 will provide insights into the adaptation to extreme haloalkaline conditions and mechanisms of reduction of some toxic oxyanyons.

### Nucleotide sequence accession number.

The draft genome sequence of *H. chromatireducens* strain has been deposited in GenBank under the accession number CP014226. The version described in this article is the first version.
